# From research to action: recommendations for Charles Bonnet syndrome care and policy

**DOI:** 10.1136/bmjophth-2024-002009

**Published:** 2025-05-02

**Authors:** Lee Jones, Jasleen K Jolly, Judith Potts, Tamsin Callaghan, Katharine Fisher, I. Betina Ip, Holly Bridge, Robin Walker, Mariya Moosajee

**Affiliations:** 1 Institute of Ophthalmology, UCL, London, UK; 2 Department of Optometry and Vision Science, University of Melbourne, Melbourne, Victoria, Australia; 3 Jolly Vision Science, Melbourne, UK; 4 Esme’s Umbrella, London, UK; 5 Royal Free London NHS Foundation Trust, London, UK; 6 Manchester Academic Health Science Centre, University of Manchester, Manchester, UK; 7 University of Oxford Nuffield Department of Clinical Neurosciences, Oxford, UK; 8 Department of Psychology, Royal Holloway University of London, Egham, UK; 9 Moorfields Eye Hospital NHS Foundation Trust, London, UK; 10 The Francis Crick Institute, London, UK

**Keywords:** Diagnostic tests/Investigation, Medical Education, Public health, Rehabilitation, Visual perception

## Introduction

 Charles Bonnet syndrome (CBS) is a curious and often unexpected outcome of sight loss. The condition is characterised by vivid, silent, visual hallucinations which range from fleeting dances of colourful patterns to fluid spectacles of faces, animals and detailed scenes. First documented over two centuries ago by Swiss philosopher Charles Bonnet,^1^ this eponymous condition has garnered renewed interest within the scientific community in recent years. This resurgence is partly due to the inclusion of CBS in the 11^th^ revision of the International Classification of Diseases (ICD), a global standard for diagnostic health information.^2^ Additionally, advancements in neuroimaging and computational modelling have opened new avenues for studying the brain and understanding the neural mechanisms underlying visual hallucinations.^3^ A growing appreciation of the impact of CBS on mental health and quality of life has prompted research into intervention development and support systems.^4 5^ Furthermore, public and professional awareness has increased through targeted outreach campaigns, media coverage and the establishment of research funding specific to CBS.^6 7^

The inaugural scientific symposium for CBS convened on November 16^th^ 2023 in London, United Kingdom, marking the eighth anniversary of CBS Awareness Day and the launch of Esme’s Umbrella, the national charity supporting people living with CBS (www.charlesbonnetsyndrome.uk). The meeting brought together researchers, clinicians, and charity leaders from across the UK working to advance understanding of the condition. The purpose was to offer a platform to discuss challenges and future priorities for CBS, a condition which remains underdiagnosed and poorly understood. The authors of this article represent the symposium attendees and bring expertise across multiple disciplines, including ophthalmology, optometry, vision science, psychology, computational and cognitive neuroscience, and the charity sector. This article compiles the major insights and conclusions from the symposium, with a focus on the contentious issues surrounding CBS.

### Refining the diagnostic criteria

Wide and/or ambiguous diagnostic criteria for the syndrome is a challenge. For example, the WHO ICD refers to hallucinations in CBS as being exclusively ‘complex’ in nature,^2^ thereby excluding those experiencing only simple ‘elementary’ hallucinations, which are the most common ophthalmological presentation. Simple hallucinations generally consist of geometric shapes and patterns, whereas complex hallucinations can involve formed images of animals, faces, people and vivid scenery. The presence of basic visual phenomena such as flashes of light are common in some ophthalmological conditions,^8^ which can be due to spontaneous activity of degenerating retinal cells. While it is important to distinguish this from CBS, referring to CBS only as involving complex hallucinations overlooks the wide range of experiences that patients can report and limits the understanding of the condition and its variability. Doing so also risks creating a gap in research, where studies may only focus on those with complex hallucinations, leading to incomplete data and a skewed understanding of how CBS manifests across different individuals. A definition of CBS which is inclusive of both simple and complex hallucinations can ensure research encompasses the full spectrum of the condition. The subsequent benefits would include better data on prevalence, risk factors, and patient experiences, which could, in turn, improve diagnostic tools and patient support services.

### Charles Bonnet syndrome (CBS) terminology

Consistent and standardised terminology is essential to facilitate clear communication. Cross-disciplinary differences are present, such as the use of ‘visual release hallucinations’ used more readily in psychiatry, a term emphasising the brain’s reaction to sensory deprivation caused by vision loss. While accurate, it is more technical and less commonly used in ophthalmic clinical practice, which may not resonate with patients or general healthcare providers. The term CBS is widely accepted and is preferred in clinical and academic settings because it precisely defines the syndrome’s relationship with visual impairment. The use of eponyms in medical nomenclature is debated, with some scholars advocating for their removal due to issues of historical accuracy, cultural bias and lack of descriptive clarity.^9^ While terms with more anatomical or functional precision might enhance understanding, they can lack the brevity and recognisability that eponyms often provide. Moreover, eponyms can serve as important historical markers that recognise the contributions of early researchers and provide a sense of continuity within medical literature. CBS is now a well-established term in clinical and patient communities, and replacing it could lead to confusion and disrupt awareness efforts. In contrast to some outdated or ethically problematic eponyms, there are presently no significant historical or moral concerns associated with Charles Bonnet himself that would justify retiring the term. In the case of CBS, preserving the eponym will help to maintain a shared language across different disciplines.

When initially discussing CBS with patients, clinicians can adopt softer language to introduce the topic. For example, structured history taking in Parkinson’s Disease uses questions such as ‘have you noticed anything unusual about your vision? Do you feel like your eyes sometimes play tricks on you? Do you ever see things that aren’t really there?’.^10^ This terminology can encourage more open and comfortable dialogue with patients, providing a natural segue into discussing CBS in greater detail. Alternative terms in use, such as ‘phantom visions’, are less formal and may unintentionally carry a connotation of something more supernatural or unsettling, adding to the stigma associated with visual hallucinations.^11^ Phantom eye syndrome is a condition causing feelings of pain or other sensations in an enucleated eye.^12^ When presenting alongside visual hallucinations, the term ‘CBS plus’ may serve as a useful descriptor to encompass both syndromes. This terminology has also been proposed where CBS co-exists with cognitive impairment in the patient.^13^ By adopting this term, clinicians and researchers can facilitate more cohesive discussions, promoting a broader understanding of the spectrum of visual and sensory experiences encountered by patients with vision loss or enucleation.

### Competing pathological process theories

The best way to develop appropriate targeted therapies is to better understand the underlying pathophysiology causing the hallucinations in CBS. This will also help to promote CBS to the wider medical community, as has happened with other diseases historically. There are currently several proposed mechanisms.^14 15^ The key feature of CBS is the loss of sensory input, so treatment of the underlying pathology will help to ease the symptoms. Other proposed factors are the imbalance in excitatory and inhibitory neurotransmitters in the visual cortex; spontaneous neural discharge within the visual brain; presence of feedback signals in the absence of feedforward signals causing complex hallucinations; changes in the blood supply for the visual brain; or hyperexcitability particularly in the ventral stream. Identifying which of these mechanisms is responsible will help to identify reliable therapeutic targets.

### Approaches to patient management

Patient management primarily relies on reassurance and education, which has been shown to mitigate the negative psychological impact of CBS.^16^ Educating patients that their symptoms are a result of visual loss, rather than indicative of a psychiatric or neurological disorder, often provides significant relief. Patients may also be comforted to learn that CBS is a common occurrence in people living with visual impairment. Besides improving vision where possible, there are limited medical approaches to treating CBS. Several therapeutic candidates have been associated with reducing or eliminating hallucinations, including antipsychotics, anticonvulsants, anti-dementia medications and selective serotonin reuptake inhibitors.^14^ However, the quality of evidence supporting these treatments is generally low, coming primarily from case reports.^14^ As such, there are no definitive medication recommendations, and patients are seldom prescribed treatment. Exploratory research suggests that inhibitory transcranial direct current stimulation can reduce the frequency of visual hallucinations,^17^ though more research is required to understand its long-term efficacy. In the absence of robust medical treatments, patients report developing their own mechanisms to cope, including behavioural exercises or ‘brain shunting’ techniques such as frequent blinking or rapid eye movements, changing light levels to increase visual input and alerting/distraction techniques.^7^ These approaches may ease hallucinations as attentional resources are redirected through the introduction of competing stimuli, allowing for temporary relief. Management strategies recommended to patients should be individualised, as their effectiveness can vary. Research from psychiatry has shown that personalised mental health support can be highly beneficial for improving patient treatment outcomes.^18^ Future research should explore the factors that influence why certain management approaches in CBS work well for some but not for others. For instance, while some patients may find interacting with their hallucinations to be effective, others may prefer strategies that involve ignoring them. Although evidence for these strategies remains largely anecdotal, their potential benefits warrant consideration for inclusion in clinical practice guidelines. Such approaches not only provide patients with practical tools for managing their symptoms, but also foster a sense of control, which can be empowering and may enhance confidence in handling future episodes.

### Requirement for clinical practice guidelines

Improved clinical protocols should be established for identifying patients with CBS, with particular attention given to standardising care across different regions and institutions. Inconsistencies exist in a number of areas including the quality of information provided to patients and variability in the interpretation and application of a CBS diagnosis. This is shown in research assessing clinical practice patterns, evidencing variability in CBS documentation and management.^19^ Prevalence estimates of CBS vary widely depending on the specific patient population studied.^20^ Recent evidence indicates a prevalence of approximately 20% among individuals with low vision;^21^ however, patients are rarely asked about visual hallucinations during routine clinical visits, leading to significant gaps in accurate prevalence data. There is a need for a simplified and harmonised framework to aid identification and management of CBS which is relevant and understandable to all healthcare providers. These guidelines should offer a structure for discussing visual hallucinations with patients in the eye clinic, with provision of recommended advice and, where necessary, referral options in cases where CBS imposes a negative impact on daily life. Such a framework should align with other consensus guidelines for patients with visual hallucinations, for instance the National Institute for Health Research (SHAPED: Study of visual Hallucinations in Parkinson’s disease, Eye disease and Dementia) which provides a unified framework for clinical management based on combined current best practice and treatment evidence.^22^ Development of CBS management guidelines should involve a diverse range of multidisciplinary stakeholders including clinicians, researchers and policymakers to ensure evidence-based practices are followed while avoiding unnecessary referral which threatens to add further strain to clinical services. Endorsement of these guidelines from bodies such as the Royal College of Ophthalmologists and the College of Optometrists would help to provide authority and legitimacy to CBS patient management, as well as increasing awareness and improving clinician attitudes towards the condition.

### Reluctance of healthcare professionals to discuss Charles Bonnet syndrome (CBS)

Although progress has been made in raising awareness, reluctance may remain among medical and allied health professionals to discuss the condition with patients, which may stem from several underlying factors. A lack of confidence in addressing the condition—either due to limited understanding or uncertainty about how to manage it effectively—acts as a significant barrier. Others may view inquiries about CBS as unnecessary, perceiving the condition as benign or one that does not warrant focused attention. Time constraints in clinical practice further exacerbate this reluctance, with clinicians already managing large patient caseloads coupled with administrative responsibilities of record-keeping and coordination of care. There have been calls for increased awareness of the risk of CBS among medical staff involved in patient care.^23^ Previous research has shown that approximately one-third of CBS patients believed that their medical professional was unsure or did not know about the condition.^16^ As the first point of contact for many patients experiencing CBS, ophthalmologists play a crucial role in early recognition and management. In that respect, presenting to a team of well-informed healthcare professionals may make the difference between patients effectively adjusting to their CBS with a high degree of resilience or being poorly equipped to self-manage the condition. Expanding education and training, particularly beyond ophthalmic professions, such as through CBS-specific modules that cover the pathophysiology, clinical presentation, differential diagnoses and management strategies, could enhance early recognition, improve referral pathways and ensure more comprehensive care for patients. Embedding CBS education into ophthalmology curricula and professional development programmes would help clinicians develop the confidence and expertise needed to recognise and discuss CBS with patients, facilitating a better understanding of the condition. There is a shared responsibility among all those working within a healthcare organisation, including executive leadership, departmental heads, healthcare providers and quality improvement teams to ensure all patients are appropriately supported, inclusive of those living with CBS.

### Outcome measures used in research

The growing body of research into CBS necessitates careful consideration regarding outcome measures. Outcome measures refer to specific metrics or criteria used to assess the effectiveness of interventions, the severity of symptoms or patient quality of life. While uniform adoption of measures is seldom possible, unity would facilitate comparability between studies and ensure that interventions and therapeutics are evaluated in a standardised way. Generally, CBS is operationalised using specific phenomenological aspects of the condition, such as duration, frequency and intensity of hallucinations. Alongside these phenomenological features, it is important to consider individual differences in appraisal and emotional response. For example, a patient can experience visually disturbing content, but may not necessarily feel concerned by the hallucination. They might appraise these experiences as harmless despite the unsettling nature of the images. Conversely, some individuals may experience relatively mundane or trivial hallucinations (eg, simple patterns or everyday objects), but their personal interpretation might provoke feelings of fear, anxiety or concern. By broadening the focus of outcome measures to include individual differences in appraisal and emotional response, researchers can gain a more holistic understanding of the condition. One approach is to assess perceived threat to physical or psychological well-being and measure whether safety-seeking behaviours are adopted, such as distraction from or interaction with the hallucination.^24^ This approach acknowledges that the same hallucination can have varying psychological impacts on different patients, depending on how they interpret and respond to their experiences.

### Conclusions

The first CBS scientific symposium served as a cornerstone event for reviewing research and policy in the UK, emphasising both the progress made and the gaps which remain. Among the key issues identified were the need for standardised outcome measures across studies, consistent and appropriate terminology, as well as the importance of exploring individual variations in the experience and appraisal of the condition. Future priorities focused on developing an interdisciplinary and harmonised approach to patient management, integrating ophthalmology, psychiatry, general practice and community services, ensuring that both the visual and emotional impact of CBS are addressed. Continued collaboration between researchers, clinicians and patient groups will be essential in advancing these efforts and shaping the future directions of research and clinical care in people with CBS.
